# The multiple faces of tuberculosis in HIV infected patients – a continuous challenge

**DOI:** 10.1186/1471-2334-14-S4-P31

**Published:** 2014-05-29

**Authors:** Raluca Ioana Dulamă, Cristina Popescu, Irina Lăpădat, Alina Lobodan, Anca Ruxandra Negru, Mihaela Rădulescu, Cătălin Tilişcan, Violeta Molagic, Raluca Năstase, Roxana Petre, Victoria Aramă

**Affiliations:** 1 National Institute for Infectious Diseases “Prof. Dr. Matei Balş”, Bucharest, Romania

## 

Tuberculosis (TB)/HIV co-infection represents a major problem in many regions of the world, including Romania. TB is a leading cause of death among people infected with HIV and HIV infection is the most important risk factor for progression from latent to active TB. TB can occur at any stage of HIV disease and its manifestations depend on the severity of immunosuppression. The proportion of extra-pulmonary tuberculosis in HIV infected patients has increased. Aim: to analyze the cases of pulmonary and extrapulmonary TB in HIV-seropositive patients monitored in Third Department of the “Matei Balş” Institute.

We performed a retrospective analysis of all HIV infected patients monitored in our clinic from 2000 to 2014 in order to establish the location of TB, the diagnosis methods, the correlation with the immune status and the outcome.

122 patients were retrospectively analyzed; from them, 18 patients were diagnosed with certain, probable or possible TB infection (14.75%). Sex ratio in TB group was M:F=1.57:1 and mean age was 39.7 years old at the moment of TB diagnosis. TB occurs at a variable level of immunosuppression (CD4 count from 6 to 460/cmm) - 4 patients (22.2%) in stage 2 - CD4=200-500/cmm and 14 patients (77.8%) in stage 3 - CD4<200/cmm. Mean CD4 count in TB group was 113.23/cmm vs. 218.33 mean nadir CD4 count in non-TB group. Pleuro-pulmonary TB accounted for only 27.7% of all cases - one pleural effusion and 4 pulmonary TB. In most of cases, TB infection was extrapulmonary (72.3%): 5 cases of meningoencephalitis (27.7%), 3 cases of disseminated TB (16.66%), 2 cases of lymph node TB (11.11%) and 3 cases with unknown location (16.66%). TB was microbiologically confirmed in only 6 cases – 33.33%, 3 by blood culture, 2 by PCR (one from CSF and one from pleural effusion) and 1 by histopathologic exam (lymph node biopsy). In 9 cases TB was probable but without bacteriologic confirmation and in 3 cases TB was possible – prolonged fever with a good outcome under anti-TB medication. Quantiferon TB was performed in only 8 cases – in 6 cases was positive, in one case was negative and in one case was undetermined. Three patients died: one patient because of disseminated TB and two patients because of other HIV-related comorbidities.

HIV infected patients developed especially extra-pulmonary TB infection. TB can occur in any stage of HIV infection. Microbiological diagnosis in TB is positive in a small number of cases.

